# Outcomes, safety and health economics of introduction of video laryngoscopy-assisted less invasive surfactant administration

**DOI:** 10.1038/s41372-024-02162-4

**Published:** 2024-11-22

**Authors:** Venkata Gupta, Barry Weinberger, Stephanie G. Galanti, Jimikumar Patel, Gangajal Kasniya, Dalibor Kurepa

**Affiliations:** 1 Department of Pediatrics, Division of Neonatal-Perinatal Medicine, Cohen Children’s Medical Center, Northwell Health, New Hyde Park, NY USA; 2 Department of Pediatrics, Division of Neonatal-Perinatal Medicine, Ochsner Children’s Hospital, Ochsner Health, New Orleans, LA USA

**Keywords:** Outcomes research, Paediatrics, Respiratory tract diseases

## Abstract

**Background:**

Less invasive surfactant administration (LISA) is associated with better outcomes than InSurE (Intubation-Surfactant administration-Extubation). Video-laryngoscopy (VL) facilitates intubation in neonates, however safety and cost-effectiveness of VL-assisted LISA have not been evaluated.

**Methods:**

We compared the outcomes of infants receiving VL-assisted LISA (*n* = 67) with a historical cohort of infants who received InSurE (*n* = 52). Secondary aims were to evaluate safety and cost-effectiveness.

**Results:**

VL-assisted LISA was associated with reduced duration of non-invasive ventilation (NIV), reduced duration of oxygen therapy, reduced composite days on NIV and mechanical ventilation (MV), and shorter NICU stay with lower hospital costs for infants ≥29 weeks GA, compared to InSurE. In the VL-assisted LISA group, 66% of the tracheal catheters were placed on the first attempt and 16% of infants displayed desaturation during placement.

**Conclusion:**

In infants ≥29 weeks GA, VL-assisted LISA reduced exposure to NIV, oxygen, NIV and MV combined, length of stay, and cost of care compared to InSurE.

## Introduction

Premature infants with respiratory distress syndrome (RDS) frequently require respiratory support and surfactant administration. Intubation followed by surfactant delivery and mechanical ventilation (MV) exposes infants to increased risk for bronchopulmonary dysplasia (BPD) [1]. Advancements in neonatal respiratory care led to the development of the InSurE technique (Intubation-SURfactant administration-Extubation) [2]. However, InSurE still requires endotracheal tube (ETT) placement with unpredictable duration of MV and inherent risks of MV-induced volutrauma. To further decrease exposure to MV and improve respiratory outcomes, “Less-Invasive Surfactant Administration (LISA)” was developed for infants breathing spontaneously on continuous positive airway pressure (CPAP) [3]. Encouraging results from the initial feasibility studies were followed by larger randomized controlled trials and meta-analyses [3–6]. Systematic reviews confirmed that LISA significantly improved outcomes for infants with RDS when compared to InSurE [7]. Currently, the European Consensus Guidelines (2019) on the management of RDS states that LISA is the preferred mode of surfactant administration for infants on CPAP [8].

Adoption of any novel technique for clinical use is challenging. It requires procedure acceptance, extensive staff training, quality assurance, safety evaluations, and continuous monitoring of the clinical outcomes. The administration of surfactant using LISA via thin catheter usually takes only 2–3 min, but it may be associated with short-term side effects such as obstructive apnea, cough, bradycardia, and desaturations. The use of sedation during LISA may increase the risk of desaturation and requirement for positive pressure ventilation (PPV) without improving the efficacy of the procedure [9]. Alternatively, nonpharmacological methods of analgesia such as positioning, holding, tucking, and sucrose solution are available. The use of video-laryngoscopy (VL) for LISA improves airway visualization, operator’s satisfaction, and increases patient safety [10, 11]. Consistent with this, a recent large, randomized control trial showed that more endotracheal intubations were successful on the first attempt using VL than direct laryngoscopy (DL) [12].

Our primary aim was to compare short- and long-term outcomes of premature infants who received surfactant via VL-assisted LISA with a historical cohort of infants who received surfactant via DL-assisted InSurE. Secondary aims were to evaluate the safety and cost effectiveness of the two methods of delivering surfactant.

## Materials and methods

### Study design, setting, and populations

This was a retrospective study of infants born between 23 0/7 and 31 6/7 weeks gestational age (GA) who did not require endotracheal intubation in the delivery room, were admitted to the NICU on bubble CPAP, and subsequently received surfactant replacement therapy for RDS in the NICU. Infants requiring intubation for clinical indications either during delivery room stabilization or in NICU, and those born with congenital anomalies of the airway were excluded. The study was conducted at two NICUs (Cohen Children’s Medical Center, New Hyde Park, NY and North Shore University Hospital, Manhasset, NY), which together are comprised of 99 beds. Both NICUs are covered by the same medical staff and use the same clinical guidelines.

We compared outcomes of premature infants who received surfactant via VL-assisted LISA (January 2021–December 2021) with infants who had received surfactant via DL-assisted InSurE prior to the introduction of LISA (January 2019–December 2020). Eligible infants were identified using the NICU electronic database. The number of infants enrolled in the InSurE cohort was limited due to data access issues related to a change in the electronic medical record (EMR) platform. Criteria for surfactant therapy were the same for both cohorts. Based on a standard NICU written guideline, surfactant was administered when FiO2 reached 30% for infants ≤28 weeks GA or 40% for infants >28 weeks during both time periods. Escalation of CPAP was based on the work of breathing and performed at the clinician’s discretion. Similarly, intubation for MV, extubation from MV to non-invasive ventilation (NIV), and further weaning of respiratory support followed generally accepted practice standards. The procedures were completed by skilled neonatologists, neonatology fellows and advanced care providers. Data on delivery room management, respiratory support, and short- and long-term outcomes were collected from the EMR. Bronchopulmonary dysplasia (BPD) was defined according to the NICHD 2018 definition [13]. Pulmonary hemorrhage was defined as an acute event characterized by respiratory deterioration with blood coming from the upper respiratory tract or the endotracheal tube [14].

### InSurE technique

Infants in the historical cohort were treated with the standard InSurE method described by Verder et al. This technique involves surfactant administration after ETT intubation using direct laryngoscopy (DL), followed by brief MV, and early extubation typically within 1–2 h [15].

### VL-assisted LISA technique

LISA was administered using strict guidelines that include patient eligibility, procedure location, contraindications, equipment, preparation instructions, VL technique, postprocedural monitoring, equipment care and documentation. Our standard LISA equipment includes neonatal VL (Peak Medical, Netanya, Israel), 16-gauge 5.25-inch sterile angiocatheter in lieu of thin catheter, marker, measuring tape and 5 mL surfactant syringe. Bubble nasal CPAP was continued during the procedure, with escalation of the CPAP level as indicated based on the work of breathing, chest x-rays and clinical condition. We did not use analgesia or sedation since VL-assisted LISA decreases the number of intubation attempts and is quicker than conventional DL-assisted LISA [12, 16]. Infants who did not tolerate the procedure, defined by oxygen saturation below 80% or heart rate below 80 beats/min for 10 s, were allowed to recover by withdrawing the VL blade or by slowing down surfactant delivery, and adjusting oxygen. If a second surfactant dose was necessary, it was delivered by LISA in infants who remained clinically stable and were breathing spontaneously.

### Health economics

Evaluation of the health care costs was performed by using the cost-consequence model described by Yao et al. [17]. This model was developed based on previously published literature to assess economic inputs that influence total NICU costs. The key input of cost per day for NICU hospitalization was estimated based on national data. The average total cost of care (cost per patient per hospital stay) was based on the average cost per day with MV and the average cost per day without MV. Total hospital costs for each infant were generated using the number of days with and without MV, and the average cost of NICU hospitalization for each cohort was calculated by dividing the sum of patient hospital costs by the number of patients in each cohort. These results were compared overall and across GA between the two cohorts. Complications, such as BPD, IVH, ROP, and pneumothorax are the main modifiers of the total cost. However, these modifiers were not included in the calculation because there was no difference in these outcomes between the cohorts. All costs were adjusted to the inflation index between the two periods as reported by the Consumer Price Index.

### Statistical analysis

Descriptive statistics provided are mean and SD for continuous variables, or frequencies and percentages for categorical variables, unless otherwise stated. Missing data were omitted from analysis. Statistical analysis included both comparisons between the entire cohorts as well as comparisons between subgroups after stratification based on GA. Each continuous variable was tested for normality within each cohort using a Shapiro-Wilk test. A Wilcoxon rank-sum test was performed for most demographic data, delivery room data, and respiratory outcomes, where normality was not assumed. Other tests include Fisher’s exact test or chi-square test for categorical variables, and Student’s *t*-test for continuous variables. The total cost of care was calculated by the evaluated cost per day with MV and the cost per day without MV. Any *p*-value less than 0.05 was considered statistically significant. Statistical analysis was performed using R Statistical Software (R Core Team 2022, V.4.2.1.).

## Results

**Fig. 1:**
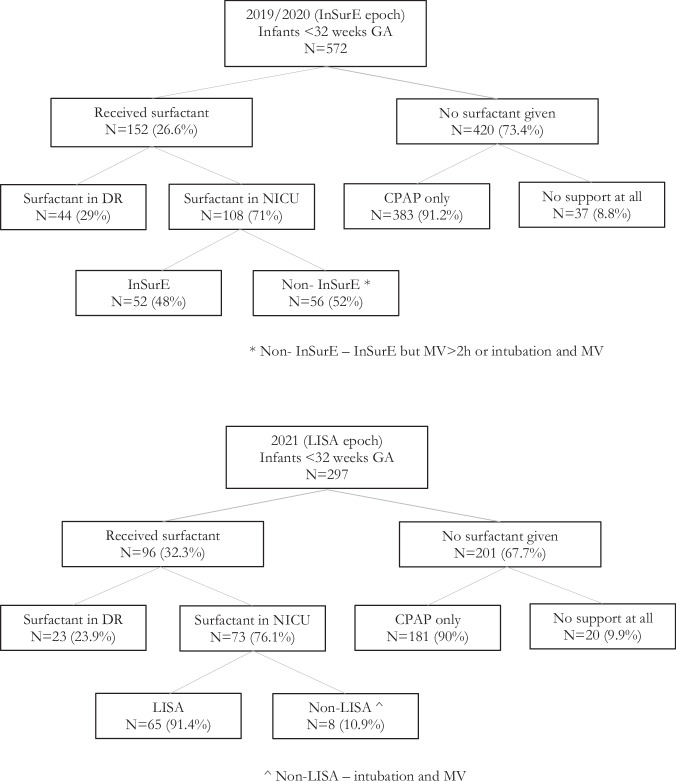
Patient inclusion flow chart.

During the InSurE epoch, 572 infants <32 weeks gestational age were admitted of whom 152 (26.6%) received surfactant, compared to 297 infants admitted during the LISA epoch of whom 96 (32.3%) received surfactant (*p* = 0.08). Among the infants who received surfactant, 44 (29%) were excluded in the InSurE group because it was administered in the delivery room for clinical indications, compared to 23 (23.9%) in the LISA group (*p* = 0.39). Thus, the rates of surfactant administration and frequencies of administration in the delivery room did not significantly change after the introduction of LISA. Among infants who received surfactant in the NICU, 56 (52%) were excluded in the InSureE group because they required either mechanical ventilation for clinical indications or prolonged mechanical ventilation (>2 h after surfactant), compared to 8 (10.9%) of infants who required mechanical ventilation for clinical indications in the LISA group (*p* < 0.0001). The lower incidence of mechanical ventilation for clinical indications in the LISA group likely reflects a *de facto* shift in the clinicians’ threshold for intubation. The flow chart for inclusion in the InSurE and LISA cohorts is shown in Fig. 1.

A total of 119 infants were included in the analysis (InSurE cohort, *N* = 52; LISA cohort, *N* = 67). In the LISA cohort, VL was applied by medical providers in 65/67 (97%) of cases and DL in 2/67 (3%) due to technical challenges with VL. Infants were stratified in 3 groups based on GA (23–25 6/7, 26–28 6/7, and 29–31 6/7 weeks). Demographic and delivery room data for both cohorts are presented in Table 1. The cohorts had similar baseline demographics, except for lower frequency of prenatal steroids and SGA in the LISA cohort and higher FiO2 requirement in the delivery room for infants in the LISA cohort born at <29 weeks GA. Overall, and in the subgroups of infants <29 weeks of GA, there was no difference in respiratory or other outcomes. However, in the LISA cohort infants born at ≥29 weeks GA had fewer days on NIV, fewer days on oxygen, and fewer composite days on NIV and MV (excluding MV related solely to InSurE) compared to the InSurE cohort. In addition, in the LISA cohort infants born at ≥29 weeks GA had shorter hospital length-of-stay and lower cost of care than infants receiving InSurE (Tables 2 and 3).

**Table 1:** Demographic and delivery room data.

Characteristic	InSurE cohort N = 52	LISA cohort N = 67	*p*-value	InSurE cohort *N* = 9	LISA cohort *N* = 13	*p*-value	InSurE cohort *N* = 24	LISA cohort N = 33	*p*-value	InSurE cohort *N* = 19	LISA cohort *N* = 21	*p*-value
Gestational Age (weeks)	-			23–25 6/7			26–28 6/7			29–31 6/7		
Prenatal Steroids	40 (77%)	39 (59%)	0.041	8 (89%)	7 (58%)	0.178	19 (79%)	22 (67%)	0.300	13 (68%)	10 (48%)	0.184
Maternal Chorioamnionitis	5 (9.6%)	6 (9.0%)	1	2 (22%)	3 (23%)	1	2 (8.3%)	1 (3.0%)	0.567	1 (5.3%)	2 (9.5%)	1
Prenatal Magnesium	42 (81%)	53 (79%)	0.822	7 (78%)	10 (77%)	1	21 (88%)	26 (79%)	0.494	14 (74%)	17 (81%)	0.712
Maternal PROM	14 (27%)	16 (24%)	0.705	4 (44%)	3 (23%)	0.376	5 (21%)	9 (27%)	0.577	5 (26%)	4 (19%)	0.712
Maternal DM	8 (15%)	13 (19%)	0.568	0 (0%)	2 (15%)	0.494	5 (21%)	5 (15%)	0.727	3 (16%)	6 (29%)	0.457
Maternal PIH	13 (25%)	27 (40%)	0.080	0 (0%)	1 (7.7%)	1	9 (38%)	16 (48%)	0.409	4 (21%)	10 (48%)	0.079
Cesarean-Section	45 (87%)	51 (76%)	0.153	6 (67%)	6 (46%)	0.415	23 (96%)	28 (85%)	0.384	16 (84%)	17 (81%)	1
Birthweight (grams)	1019 ± 397	1069 ± 414	0.500	746 ± 112	685 ± 132	0.258	864 ± 245	956 ± 263	0.181	1346 ± 429	1484 ± 383	0.290
Male	34 (65%)	38 (57%)	0.337	5 (56%)	5 (38%)	0.666	13 (54%)	21 (64%)	0.472	16 (84%)	12 (57%)	0.062
SGA	11 (21%)	5 (7.5%)	0.030	0 (0%)	0 (0%)	1	7 (29%)	4 (12%)	0.173	4 (21%)	1 (4.8%)	0.172
5-min Apgar Score Median (IQR)	8 (7–9)	8 (7–9)	0.953	8 (7–8)	8 (7–8)	0.971	8 (8–9)	8 (7–8)	0.222	8 (6–9)	8 (8–9)	0.165
Maximum CPAP in DR	5.9 ± 1.2	5.7 ± 0.7	0.747	5.3 ± 0.7	5.7 ± 0.6	0.143	6.2 ± 1.5	5.9 ± 0.7	0.972	5.9 ± 0.9	5.5 ± 0.7	0.140
Max FiO2 in DR	56.1 ± 31.6	64.9 ± 30.3	0.097	31.4 ± 10.2	63.3 ± 33.9	0.038	48.8 ± 29.8	69.5 ± 30.9	0.013	77.1 + −28.4	58.9 + −27.0	0.071

*CPAP* continuous positive airway pressure, *DM* diabetes mellitus, *DR* delivery room, *FiO2* fraction of inspired oxygen, *InSurE* intubation–surfactant administration–extubation, *IQR* interquartile range, *LISA* less-invasive surfactant administration, *PIH* pregnancy induced hypertension, *PROM* premature rupture of membranes, *SGA* small for gestational age.

**Table 2:** Respiratory outcomes.

Characteristic	InSurE cohort *N* = 52	LISA cohort *N* = 67	*p*-value	InSurE cohort *N* = 9	LISA cohort N = 13	*p*-value	InSurE cohort *N* = 24	LISA cohort *N* = 33	*p*-value	InSurE cohort *N* = 19	LISA cohort *N* = 21	*p*-value
Gestational Age (weeks)	-			23–25 6/7			26–28 6/7			29–31 6/7		
Caffeine	50 (96%)	64 (96%)	1	9 (100%)	13 (100%)	1	24 (100%)	33 (100%)	1	17 (89%)	18 (86%)	1
FiO2 before 1st surfactant (%)	45.9 ± 18.9	53.2 ± 18.7	0.073	32.0 ± 11.7	58.3 ± 21.7	**0.002**	45.5 ± 14.7	54.0 ± 17.8	0.155	52.8 ± 23.2	48.8 ± 18.3	0.420
Time to 1st surfactant (hours)	10.9 ± 17.0	10.6 ± 11.9	0.361	11.5 ± 18.1	3.9 ± 4.9	0.229	6.9 ± 11.5	10.3 ± 12.0	0.098	15.8 ± 21.4	15.3 ± 13.1	0.364
NIV (days)	52.5 ± 37.8	49.5 ± 36.5	0.3	59.0 ± 28.2	63.2 ± 43.4	0.3	59.5 ± 38.1	63.7 ± 30.8	0.3	40.7 ± 39.9	18.5 ± 16.9	0.014
MV (days)^a^	10.5 ± 19.3	5.2 ± 14.8	0.085	17.4 ± 13.9	18.8 ± 29.6	0.3	9.5 ± 14.8	2.8 ± 5.4	0.2	8.5 ± 25.6	0.6 ± 1.8	0.12
O2 therapy (days)	56.3 ± 53.6	42.6 ± 40.8	0.080	79.0 ± 36.3	66.9 ± 50.1	0.3	61.8 ± 49.9	54.5 ± 35.6	0.3	38.6 ± 61.3	8.9 ± 12.4	0.038
NIV + MV (days)^a^	63.1 ± 50.8	53.6 ± 40.9	0.14	76.4 ± 39.1	77.0 ± 55.4	0.5	68.1 ± 47.9	66.5 ± 30.6	0.5	50.4 ± 58.3	18.9 ± 16.7	0.004
MV in first 72 h^a^	16 (31%)	17 (25%)	0.3	5 (56)	6 (46)	0.5	8 (33)	9 (27)	0.3	3 (16)	2 (9.5)	0.3
Any MV^a^	23 (44%)	26 (39%)	0.3	7 (78)	10 (77)	0.5	11 (46)	13 (39)	0.3	5 (26)	3 (14)	0.2
Pneumothorax	5 (9.6%)	6 (9.0%)	1	1 (11%)	3 (23%)	0.616	1 (4.2%)	2 (6.1%)	1	3 (16%)	1 (4.8%)	0.331
Pulmonary Hemorrhage	1 (1.9%)	1 (1.5%)	1	0 (0%)	1 (7.7%)	1	0 (0%)	0 (0%)	1	1 (5.3%)	0 (0%)	0.475
Any BPD	26 (50%)	33 (49%)	0.936	6 (67%)	8 (62%)	1	13 (54%)	22 (67%)	0.339	7 (37%)	3 (14%)	0.148
Moderate to Severe BPD	13 (25%)	16 (24%)	0.888	2 (22%)	6 (46%)	0.380	7 (29%)	9 (27%)	0.875	4 (21%)	1 (4.8%)	0.172

*BPD* bronchopulmonary dysplasia, *FiO2* fraction of inspired oxygen, *InSurE* intubation–surfactant administration–extubation, *LISA* less-invasive surfactant administration, *NIV* non-invasive ventilation, *MV* mechanical ventilation.

^a^Indicates MV is not related to the InSurE technique itself.

**Table 3:** Other outcomes.

Characteristic	InSurE cohort *N* = 52	LISA cohort *N* = 67	*p*-value	InSurE cohort *N* = 9	LISA cohort *N* = 13	*p*-value	InSurE cohort *N* = 24	LISA cohort *N* = 33	*p*-value	InSurE cohort *N* = 19	LISA cohort *N* = 21	*p*-value
Gestational Age (weeks)	-			23–25 6/7			26–28 6/7			29–31 6/7		
IVH > grade II	3 (5.8%)	7 (10%)	0.510	1 (11%)	3 (23%)	0.616	1 (4.2%)	3 (9.1%)	0.631	1 (5.3%)	1 (4.8%)	1
NEC ≥ stage II	2 (3.8%)	3 (4.5%)	1	0 (0%)	1 (7.7%)	1	1 (4.2%)	2 (6.1%)	1	1 (5.3%)	0 (0%)	0.475
PDA Requiring Treatment	16 (31%)	20 (30%)	0.914	8 (89%)	7 (54%)	0.165	7 (29%)	13 (39%)	0.424	1 (5.3%)	0 (0%)	0.475
ROP > stage II	6 (12%)	10 (15%)	0.591	2 (22%)	3 (23%)	1	3 (13%)	7 (21%)	0.494	1 (5.3%)	0 (0%)	0.475
Length of Stay (days)	79.9 ± 46.9	70.1 ± 41.4	0.252	101.8 ± 42.3	85.4 ± 62.4	0.471	80.0 ± 43.0	84.4 ± 31.3	0.502	69.4 ± 52.2	38.1 ± 13.7	0.003
Death	7 (13%)	7 (10%)	0.613	1 (11%)	5 (38%)	0.333	4 (17%)	1 (3.0%)	0.151	2 (11%)	1 (4.8%)	0.596

*InSurE* intubation–surfactant administration–extubation, *IVH* intraventricular hemorrhage, *LISA* less-invasive surfactant administration, *NEC* necrotizing enterocolitis, *PDA* patent ductus arteriosus, *ROP* retinopathy of prematurity.

**Fig. 2:**
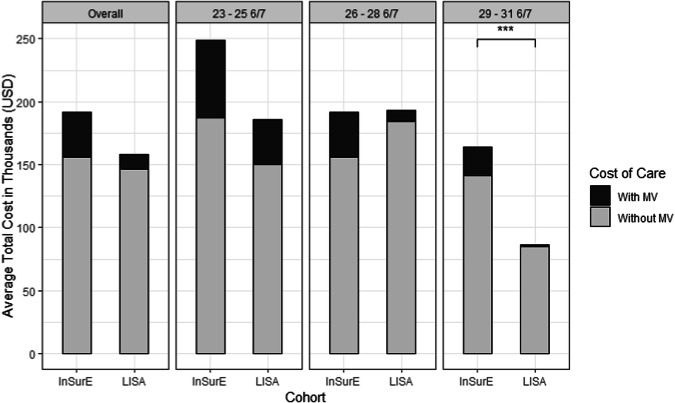
Average cost of care. Graph shows the average total cost of care (US Dollars) for both the entire InSurE and LISA cohorts and stratified by gestational ages. The average total cost of care was calculated by the sum of the evaluated average cost per day with mechanical ventilation (darker gray bars) and the average cost per day without mechanical ventilation (lighter gray bars). ***p < 0.001. (Overall) InSurE (*n* = 52) $191,455.27 ± $114,923.42; LISA (*n* = 67) $158,434.13 ± $92,208.71. (23-25 6/7) InSurE (*n* = 9) $249,195.89 ± $102,748.89; LISA (*n* = 13) $185,798.92 ± $133,091.50. (26-28 6/7) InSurE (*n* = 24) $191,779.17 ± $108,031.78; LISA (*n* = 33) $193,368.85 ± $73,309.45. (29-31 6/7) InSurE (*n* = 19) $163,695.32 ± $123,836.44; LISA (*n* = 21) $86,596.62 ± $30,201.09. InSurE intubation–surfactant administration–extubation, LISA less-invasive surfactant administration, MV mechanical ventilation.

Of infants receiving LISA, 66% (44/67) had the catheter placed successfully in the airway on the first attempt. Sixteen percent (11/67) of them had accompanying episodes of desaturation and bradycardia. The cost consequence analysis demonstrated significantly lower hospital costs for infants ≥29 weeks GA in the LISA cohort ($86,596 ± 30,201 vs $163,695 ± 123,836, Fig. 2).

## Discussion

### InSurE vs LISA clinical outcomes

We found that VL-assisted LISA was associated with reduced duration of NIV, reduced duration of O2 therapy, and reduced composite days on NIV and MV in infants >29 weeks GA, compared to InSurE. In addition, in this GA group NICU length-of-stay and hospital costs were lower. Thus, our findings are consistent with recent meta-analyses showing that LISA is advantageous compared to other methods with regard to short term outcomes [7]. In contrast, we did not find any improvement in the long-term outcomes of BPD or mortality in the LISA group. Although some previous controlled trials have found that LISA is associated with lower rates of BPD or death, this association is inconsistent in published reports [3–6]. Our ability to detect differences in long-term changes in clinical outcomes or morbidity was likely reduced because of the small size of our cohorts. In addition, the LISA cohort had a higher severity of initial respiratory illness than the InSurE cohort, as reflected in the higher maximal FiO2 that they received in the delivery room. Likewise, fewer infants in the LISA cohort received steroids prenatally and fewer were small for gestational age. These differences between the groups suggest that the benefits of LISA may be greater than we report.

Also, it is important to note that our study reports on outcomes during the first year that we performed LISA. Therefore, it is possible that the administration of LISA was delayed because of uncertainty by providers. Consistent with this, we found that 61% of infants received surfactant via LISA at >1 h of reaching the treatment threshold. Active quality improvement interventions are in place to improve the timing of LISA administration, which may improve long-term outcomes.

### VL-assisted LISA safety

VL offers improved airway visualization (enhanced lighting, capability to zoom the image, tip of the camera at the tip of the VL blade compared with the operator’s eye being several inches from the field of interest) and procedure comfort compared to the standard laryngoscopy, especially for novice intubation operators [16, 18, 19]. Our general experience parallels recent research findings where VL improved targeted guidance during catheter placement. However, the safety of the VL-assisted LISA has not been widely evaluated. In a retrospective study, VL-assisted LISA did not independently decrease the number of intubation attempts, but it decreased the number of tracheal intubation-associated events [20]. Other studies described VL as safe procedure that decreases the number of intubation attempts in infants [21]. Even though our data encompass a period during the first year of the introduction to VL-assisted LISA, we found that the number of catheter-placement attempts and procedure-associated events was similar to those previously described for DL-assisted LISA [5, 6, 22].

### Health economics

NICU care in the US is expensive but highly cost-effective [23]. The cost per infant is inversely related to the GA, but the overall cost of care is skewed towards moderately premature infants (29–32 weeks GA), who comprise the majority of NICU admissions [24]. In our study, the main contributors to the cost of NICU care, days on MV and LOS, were significantly higher in the InSurE cohort across all GA ages and in the subgroup of infants 29–32 weeks GA, respectively. This translates into substantial health economics advantages in the LISA cohort.

### Study limitations and strengths

The limitations of our study include possible issues with generalizability beyond NICUs with similar size and 24-hour in-house staffing by at least two trained operators. Unfortunately, we could not provide more historical controls due to the change in our EMR system. We interpret the secondary outcome in the GA subgroups with caution due to the small sample sizes across the subgroups. It is also important to note that the lower incidence of exclusion because of MV for clinical indications in the LISA group likely reflects a change in the clinicians’ threshold for intubation. While this represents a benefit of LISA, the exclusion of ventilated patients from the InSurE group likely made it more difficult to detect improvements in other endpoints. Exclusion of infants with more severe respiratory disease from the InSurE group may have impaired our ability to detect additional benefits from LISA. The main strength of our study is the large number of low GA infants who were treated with VL-assisted LISA. Also, all operators completed robust staff training, with detailed guidelines on LISA equipment and techniques, so we believe that the quality of VL-assisted LISA was uniform. We maintained vigorous, periodic competence checks, and comprehensive safety and quality control.

## Conclusions

VL-assisted LISA compared to InSurE is associated with reduced exposure to NIV, oxygen therapy, and combined days on NIV and MV in infants >29 weeks of GA. Importantly, it considerably decreases the length of stay in this stratum of infants, and therefore the overall neonatal cost of care. VL-assisted LISA is safe and well-accepted by NICU staff when introduced with written guidelines, intensive skills-development, and robust quality assurance.

## Data Availability

The raw data used in this study are available in REDCap database. Standardized data entry sheets were created and have been retained using REDCap (PID 20835) electronic data capture instruments hosted at Northwell Health servers. Due to the privacy issues data are subject to Northwell Health data sharing policies and on request from the corresponding author.
